# How do medical students learn conceptual knowledge? High-, moderate- and low-utility learning techniques and perceived learning difficulties

**DOI:** 10.1186/s12909-022-03283-0

**Published:** 2022-04-06

**Authors:** Anne Franz, Sebastian Oberst, Harm Peters, Ralph Berger, Ronja Behrend

**Affiliations:** 1 Charité – Universitätsmedizin Berlin, Dean’s Office of Study Affairs, Dieter Scheffner Center for Medical Education and Educational Research, Charitéplatz 1, 10117 Berlin, Germany; 2 Charité – Universitätsmedizin Berlin, Dean’s Office of Study Affairs, Department of Quality Assurance, Charitéplatz 1, 10117 Berlin, Germany; 3 Charité – Universitätsmedizin Berlin, Dean’s Office of Study Affairs, Semester Coordination, Charitéplatz 1, 10117 Berlin, Germany

**Keywords:** Learning techniques, Learning difficulties, Medical students

## Abstract

**Background:**

Acquiring medical knowledge is a key competency for medical students and a lifelong requirement for physicians. Learning techniques can improve academic success and help students cope with stressors. To support students’ learning process medical faculties should know about learning techniques. The purpose of this study is to analyse the preferred learning techniques of female and male as well as junior and senior medical students and how these learning techniques are related to perceived learning difficulties.

**Methods:**

In 2019, we conducted an online survey with students of the undergraduate, competency-based curriculum of medicine at Charité – Universitätsmedizin Berlin. We chose ten learning techniques of high, moderate and low utility according to Dunlosky et al. (2013) and we asked medical students to rate their preferred usage of those techniques using a 5-point Likert scale. We applied t-tests to show differences in usage between female and male as well as junior and senior learners. Additionally, we conducted a multiple regression analysis to explore the predictive power of learning techniques regarding perceived difficulties.

**Results:**

A total of 730 medical students (488 women, 242 men, M_age_ = 24.85, SD = 4.49) use three techniques the most: ‘highlighting’ (low utility), ‘self-explanation’ (moderate utility) and ‘practice testing’ (high utility). Female students showed a significantly higher usage of low-utility learning techniques (t(404.24) = -7.13, *p* < .001) and a higher usage of high-utility learning techniques (t(728) = -2.50, *p* < .05) than male students (M = 3.55, SD = .95). Compared to junior students (second to sixth semester; M = 3.65, SD = .71), senior students (seventh semester to final clerkship year; M = 3.52, SD = .73) showed a lower use of low-utility learning techniques (t(603) = 2.15, *p* < .05). Usage of low-utility techniques is related to more difficulties (β = .08, t(724) = 2.13, *p* < .05). Usage of moderate-utility techniques is related to less learning difficulties (β = -.13, t(599) = -3.21, *p* < .01).

**Conclusions:**

Students use a wide range of low-, moderate- and high-utility learning techniques. The use of learning techniques has an influence on the difficulties perceived by students. Therefore, they could benefit from knowing about and using high-utility learning techniques to facilitate their learning. Faculties should inform their students about effective learning and introduce them to useful learning techniques.

**Supplementary Information:**

The online version contains supplementary material available at 10.1186/s12909-022-03283-0.

## Introduction

Due to constant progress in medicine, acquiring, expanding and questioning knowledge is a lifelong key competency for every physician [1]. Medical students learn these skills within complex curricula, which is either traditional or integrated, with various types of teaching and learning formats and forms of assessment. All curricular characteristics have in common that medical students are trained to acquire knowledge, skills and attitudes to be optimally prepared for their clinical practice as physicians. The greatest stressors in medical students’ learning are the workload at medical schools, performance pressure and study examinations [2, 3]. Medical faculties should know about students’ learning habits to be able to support students’ ability to deal with stressors, solidify the ability to learn and find appropriate effective techniques to facilitate students’ successful graduation.

Academic success is linked to a complex system of individual and external learning factors such as students’ motivation, self-regulation, learning effort, environmental interactions, and financial and social support [4–6]. Learning techniques (e.g., early revision of topics; prioritization of learning needs; deeper learning; mind mapping) comprise one factor that influences the academic success of high-achievement medical students [7]. Successful students (mostly or all A’s) more likely physically attend classes, tend to study six to eight hours a day and review lecture material on the same day [6]. Kumar et al. [8] identified self-reported study habits of students with a higher score at the USA Medical Licensing Examination. They found that those who estimated completing more than 2000 practice questions also obtained higher scores. Previous research clusters learning into deep, surface, monitoring and strategic approaches. Deep approaches refer to an intention to understand (e.g., relating ideas), while surface approaches include unreflective learning (e.g., memorizing, reproducing). Monitoring approaches are related to a deep approach but describe a metacognitive aspect of learning. A strategic approach covers the organization of study (e.g., time management) and effort management (e.g., concentration) [9]. Surface learning and poor time management are associated with worse academic performance at medical school, while strategic and deep learning approaches show a significant, positive correlation with academic success [10–13]. The results of learning techniques used among medical students differ in the literature. Chonkar et al. [14] reported that medical students’ predominant learning approaches are of deep and strategic nature and that a minority uses the surface approach for learning. However, it is worth taking a closer look at the level of study: graduated students are more likely to adopt the approach of study monitoring, while undergraduate students increasingly use a surface approach for learning [14]. Samarakoon et al. [15] reported that first- and final-year students and postgraduate students mostly use surface approaches. Learning approaches can also be affected by the age of a medical student but not by gender [16]. First-year medical students report preferring visual (demonstrations, photographs, and diagrams) and sequential learning styles (building up thoughts stepwise and linear instead of holistically thinking) [17].

Derived from cognitive and educational psychology, Dunlosky et al. [18] developed evidence-based recommendations for ten learning techniques regarding utility (low, moderate, high). The authors based their choice for the ten learning techniques on a literature review on empirical evidence and on students’ reports on their use of learning techniques. The authors evaluated the benefits of these techniques across four categories of variables: learning conditions, student characteristics, materials, and criterion tasks. Learning conditions include aspects of the learning environment in which the technique is implemented (e.g., group vs. individual learning; reading vs. listening). Student characteristics include variables such as age, interests, verbal ability, and prior knowledge. Materials include maps, diagrams, science definitions and lecture content. Criterion tasks include different aspects, such as cued or free recall, problem solving, and comprehension. All ten techniques can be used by students without assistance and supervision. The techniques include practice testing and distributed practice (high utility); elaborative interrogation, self-explanation and interleaved practice (moderate utility); and summarization, highlighting (or underlining), keyword mnemonic, imagery use for text learning and rereading (low utility) [18].

Various studies have explored the learning techniques defined by Dunlosky et al. (2013). D’Antoni et al. [19] described how to learn information from a clinical anatomy textbook using effective strategies that have been experimentally validated through cognitive and educational psychology. The authors suggested embracing learning techniques such as ‘distributed practice’, ‘practice testing’ and ‘successive relearning’ for learning anatomy. The following two publications from Piza et al. [20] and Lerchenfeldt & Nyland [21] indicated a lack of understanding of students for using the effective and useful learning techniques introduced by Dunlosky et al. [18]. Piza et al. [20] found that students of health professions use learning techniques that violate evidence-based principles. A majority of teachers in their study (89%) stated that they recommend that students use highly useful ‘self-testing’, but only 34% of their students actually reported using this learning technique. Additionally, a large number of the teachers recommended nonefficient learning techniques such as ‘repeated reading’ (31%) and ‘highlighting’ (31%) to their students. Lerchenfeldt & Nyland [21] analysed 2-year medical student preferences for different learning techniques during their psychopathology course and evaluated the efficacy of those learning techniques by correlating student use with academic performance. They found that students used half of their general study time focusing on the lowest-utility learning techniques (highlighting, keywords, re-reading, and summarization). There are differences in the use of techniques depending on students’ learning in general or for exam preparation. The high-utility techniques ‘practice testing’ and ‘self-elaboration’ were only used by less than one-third of students in their general study time, although almost half of the pre-exam study time was spent on practice testing.

To better prepare students for clinical practice, medical schools have reformed their curricula and changed learning to integrated, interdisciplinary approaches with new methods of teaching and learning [17, 22, 23]. In integrated curricula, a connection between preclinical and clinical courses combines conceptual and procedural knowledge. Conceptual knowledge represents the ‘what’, e.g., learning facts from a textbook is the basis for applying procedural knowledge, thus representing the ‘how’ and ‘why’ for solving medical problems [24]. Students learn, e.g., anatomy or physiology (the ‘what’) together with clinical diseases and patient cases (the ‘how’ and ‘why’) in learning formats such as problem-based learning. The Harvard Medical School statutes an example when changing the curriculum to an integrated approach. In the new curriculum educators focus on how students learn and students are facilitated in their ability to apply knowledge  in the clinical practice [23].

Both medical schools with traditional curricula and those with integrated curricula set high learning requirements (e.g., medical college admission test; A levels) for selection, which is a good predictor of performance in undergraduate medical training [10]. Therefore, it is reasonable that medical students have already required basic learning skills before they enter medical school. However, medical studies require a high level of commitment and hard learning, which can be overwhelming and challenging [2, 3].

The purpose of this explorativestudy is to examine the learning habits of medical students at our institution with an integrated study program. We analysed 1) what learning techniques medical students in an integrated curriculum prefer and if there are differences according to gender (female vs. male) or study progress (junior vs. senior students) and 2) how learning techniques of low, moderate and high utility are related to perceived learning difficulties.

## Methods

### Setting

We conducted the study with undergraduate students of the Modular Curriculum of Medicine (MCM), an integrated, modular, outcome-oriented and competency-based medical study program at the Charité – Universitätsmedizin Berlin (Charité). The MCM consists of 40 modules spanning ten semesters and culminating in a final clerkship year, which results in a total of six years, with 330 students enrolled each semester [25]. In addition to other forms of assessment, it is mandatory for the students to pass multiple-choice exams at the end of each semester. We asked all students at Charité Berlin to complete a standardized online questionnaire about their use of learning techniques and the difficulties they experience during their studies. The questionnaire was part of an online survey used to evaluate the MCM.

### Sample and study protocol

From January to March 2020, all students of the MCM from the second semester up to those in their final clerkship year (*N* = 3431) were invited to take part in the evaluation at the end of the semester. We created the questionnaire using the survey software evasys (evasys GmbH Lüneburg, Germany). An invitation was sent out via e-mail directing the students to the online survey. After the invitation, we sent further e-mails to remind the students about the survey. To support the recruiting process, Facebook groups and posters on the campus were used to call for participation in the voluntary survey. Informed consent of all students was collected, and we used pseudonyms for the students. All data was saved in evasys after the students completed the survey. The study was approved by the ethics committee of the Charité (No. EA1/300/21).

### Questionnaire on learning techniques

The extent to which students use certain learning techniques during their studies was measured with a standardized questionnaire consisting of ten items describing common learning techniques (elaborative interrogation, self-explanation, summarization, highlighting/underlining, keyword mnemonic, imagery for text, rereading, practice testing, distributed practice and interleaved practice) as defined by Dunlosky et al. [18]. Students had to rate on a 5-point scale (1 = strongly agree; 5 = strongly disagree) to what extent they were using each technique (see Additional file 1: Appendix 1). Giving multiple answers was possible. All items were recoded to indicate that high values represent high agreement ratings. The answer options of strongly agree and agree were taken together to calculate the percentage of an overall agreement of the students on their use of the learning techniques. For further analyses, all learning techniques were aggregated into techniques of low utility (summarization, highlighting/underlining, keyword mnemonic, imagery for text and rereading), moderate utility (elaborative interrogation, self-explanation and interleaved practice) and high utility (practice testing and distributed practice), as recommended by Dunlosky et al. [18].

### Questionnaire on difficulties during studies

To capture the students’ difficulties during their studies, we calculated the mean of four items on a 5-point scale (1 = very large difficulties; 5 = no difficulties). All items were recoded to indicate that high values represent a high level of difficulties (see Table 1).

**Table 1:** Items of the standardized questionnaire to capture the difficulties students experience during their studies

What causes you difficulties in your daily study routine?
1 = very large difficulties; 2 = large difficulties; 3 = moderate difficulties; 4 = little difficulties; 5 = no difficulties
Performance requirements during studies
To prepare exams efficiently
The lack of freedom to close knowledge gaps
To master the amount of learning content

### Data analysis

After data acquisition, we downloaded the data and transferred the dataset into IBM SPSS Statistics for Windows, version 25 (IBM Corp., Armonk, N.Y., USA). Descriptive and inferential analyses were performed in SPSS.

We calculated descriptive statistics for each of the ten learning techniques separately according to the extent of use reported by the students. We used two independent samples t-tests to compare the use of learning techniques aggregated according to utility categories (high, moderate, low) with both gender and study progress as grouping variables in one t-test each. To test whether learning techniques of high, moderate or low utility (independent variables) predicted difficulties experienced by the students during their studies (dependent variable), we utilized a multiple regression analysis and controlled for gender and study progress by entering both factors as additional independent variables.

## Results

### Participants

A total of 824 students responded (response rate: 24%), of which 94 students were excluded from the analysis because of missing relevant data. This resulted in a dataset of 730 students (488 women, 242 men, M_age_ = 24.85, SD = 4.49) who completed the questionnaire. The final dataset contained 404 students categorized as junior students (second to sixth semester) and 326 senior students (seventh semester to final clerkship year).

### The overall use of learning techniques

Table 2 describes the students’ usage of all ten common learning techniques according to Dunlosky et al. [18]. One technique in each utility category (low, moderate & high utility) reached comparably high agreement: ‘highlighting/underlining’ (low utility, 74%), ’self-explanation’ (moderate utility, 74%) and ‘practice testing’ (high utility, 76%) reached the highest usage, while the students’ lowest usage level was with ‘interleaved practice’ (moderate utility, 25%). It was notable that ‘distributed practice’ (47%) showed the second lowest usage even though it is considered a high-utility learning technique according to Dunlosky et al. [18]. ‘Keyword mnemonic’ as a low-utility learning technique reached the same level of usage (47%). When classified into utility groups, students reported to use low-utility learning techniques (‘summarization’, ‘highlighting/underlining’, ‘keyword mnemonic’, ‘imagery for text’ and ‘rereading’) with an average of 60% and high-utility learning techniques (‘practice testing’ and ‘distributed practice’) with an average of 62%. With an average of 53%, students used less learning techniques of moderate utility (‘elaborative interrogation’, ‘self-explanation’ and ‘interleaved practice’).

**Table 2:** The students’ level of agreement on the use of common learning techniques according to Dunlosky et al. [18], *n* = 730

Learning technique	Usage (in %)	Usage according to utility in (%)
Summarization	52	Low-utility learning techniques: 60
Highlighting/Underlining	74	
Keyword Mnemonic	47	
Imagery for Text	54	
Rereading	71	
Elaborative Interrogation	60	Moderate-utility learning techniques: 53
Self-explanation	74	
Interleaved Practice	25	
Practice Testing	76	High-utility learning techniques: 62
Distributed Practice	47	

### The use of learning techniques according to gender and study progress

We used scores aggregated according to utility (low, moderate, high) to picture the differences in the use of learning techniques according to gender. Compared to male students (M = 3.32, SD = 0.78), a t-test revealed that female students (M = 3.73, SD = 0.63) showed a significantly higher usage of low-utility learning techniques (t(404.24) = -7.13, *p* < 0.001), which represented a medium effect (d = -0.60, 95% CI [-0.76; -0.44]) according to Cohen [26]. Furthermore, a t-test indicated that female students (M = 3.73, SD = 0.90) showed a significantly higher usage of high-utility learning techniques (t(728) = -2.50, *p* < 0.05) than male students (M = 3.55, SD = 0.95), which represented a small effect (d = -0.20, 95% CI [-0.35; -0.04]) according to Cohen [26]. There was no significant difference in the usage of moderate-utility learning techniques between female and male students.

We used scores aggregated according to utility (low, moderate, high) to capture the differences in the use of learning techniques according to study progress. Compared to junior students (M = 3.65, SD = 0.69), a t-test indicated that senior students (M = 3.52, SD = 0.73) showed a significantly lower usage of low-utility learning techniques (t(728) = 2.38, *p* < 0.05), which represented a small effect (d = 0.18, 95% CI [0.03; 0.32]) according to Cohen [26]. Further t-tests indicated no significant difference in the usage of moderate- and high-utility learning techniques between junior and senior students.

### Learning techniques, gender and study progress as predictors of difficulties during studies

Additionally, we tested whether all the discussed concepts (learning techniques, gender, study progress) predicted difficulties experienced by students during their studies. Multiple regression analysis revealed that all the factors together predicted 10% of the variance (R^2^ = 0.10, F(5, 724) = 16.37, *p* < 0.001), which represented a low-to-medium prediction of variance of the difficulties during studies according to Cohen [26]. Higher usage of low-utility learning techniques significantly predicted more difficulties during studies (β = 0.08, t(724) = 2.13, *p* < 0.05). Higher usage of moderate-utility learning techniques significantly predicted less difficulties during studies (β = -0.11, t(724) = -2.97, *p* < 0.01). Gender significantly predicted difficulties during studies since female students experienced significantly more difficulties during studies than did male students (β = 0.11, t(724) = 2.98, *p* < 0.01). Study progress significantly predicted difficulties during studies since senior students experienced significantly less difficulties during studies than junior students (β = -0.24, t(724) = -6.84, *p* < 0.001).

## Discussion

This study provides insight into the role of learning techniques and students’ use of these learning techniques to master the requirements of the integrated medical curriculum at Charité. The results showed that students use a wide range of different learning techniques along different utility categories (low, moderate and high utility). The four most commonly used learning techniques are distributed across the three utility groups. Students use the basic learning techniques of ‘repeated reading’ and ‘underlining’, possibly to become fundamentally familiar with large amounts of material from textbooks. In our integrated curriculum, problem-based learning is a core element, which might explain why ‘self-explanation’ is frequently used by students. In contrast to the results of other publications [20, 21], in our study, ‘practice testing’ was highly used by students. In Germany, an established and commercially available online system centred around multiple-choice questions from medical state examinations exists as a tool for ‘practice testing’, which could explain the high usage value of this particular learning technique. Moreover, only half of the students (45%) used the second high-utility learning technique, namely, ‘distributed practice’, possibly because students tend to use their majority of learning time prior to assessments (i.e., ‘cramming’) [18].

The results of this study show that female students use a wider range of learning techniques and they report more learning difficulties. This is consistent with the findings of Hill et al. (2018), which show that female students report more difficulties with managing academic workload than male students [2]. Furthermore, female students are at higher risk for mental illness and burnout during medical studies [27]. Therefore, faculties should pay special attention to the needs of female students. On the one hand, they should give support in learning techniques, but also provide offers to improve mental health.

The results show that senior students agree less about the use of low-utility learning techniques. It is possible that low-utility learning techniques are not sufficient in senior studies due to the performance demands and amount of material. This possibility goes hand in hand with the learning progression from easy to hard learning content, which is provided in the study program. It is possible that the low-utility learning techniques are sufficient at the beginning but then become no longer sufficient in the progress of students’ study. One explanation is that those who used low-utility learning techniques either failed in their studies or adapted more useful ways of learning.

Regarding the difficulties experienced by students, we observed that a higher level of agreement on the use of low-utility techniques predicted higher levels of perceived difficulties. At the same time, moderate-utility learning techniques predicted fewer perceived difficulties. This result is logically comprehensible and shows that the use of learning techniques of different utility categories influenced the perceived difficulties. One would assume that the high-utility learning techniques would be associated with even fewer difficulties, which was not found in our data. An explanation for this outcome could be that the two high-utility learning techniques show different characteristics than those of the low- and moderate-utility techniques. Both high-utility learning techniques require that students use other learning techniques either before or in combination. ‘Practice testing’ is a learning technique that helps students recall information that they already learned before with another technique (e.g., ‘practice testing’ is used after acquiring knowledge due to ‘rereading’ and ‘summarization’). On the other hand, ‘distributed practice’ (= learning over time) aims to organize the time management of learning. Therefore, ‘distributed learning’ must also be linked to another learning technique, e.g., distribute ‘practice testing’ in episodes over time.

Study progress is the most important predictor associated with perceived difficulties. Senior students may experience fewer difficulties because they have overcome initial difficulties in the progress of their studies. The reasons for this may be that they have learned coping strategies, learned to take advantage of offers of help and perhaps also learned to learn better by using the right learning techniques for themselves individually. Universities could create individual learning opportunities for their students that are adapted to the curriculum. One example is the changed curriculum of the Harvard Medical School which is based on best evidence principles from cognitive science, e.g. retrieval practice (test-enhanced learning), spaced practice (revisiting material learned over time) and interleaving (interspersing different materials) [23]. Another example is motivating students to use the highly useful learning technique practice testing by including multiple choice questions at the beginning or at the end of courses. But of course, resources of medical schools are limited. Therefore, universities should provide support and information about learning opportunities to the students, but in the end the decision and responsibility which learning techniques are used lies on the students themselves.

### Limitations

The single-centre study had a large sample (*N* = 3431) and an acceptable response rate (24%). We only surveyed students from one integrated medical curriculum; thus, the transferability to other faculties is limited.

One should take into account that some of the learning techniques of Dunlosky et al. [18] depend on each other (e.g., using ‘practice testing’ without learning something before using another technique). This could have influenced the answers given by students.

Furthermore, it is likely that students also use other learning techniques, which were not asked in this study. Learning techniques according to Dunlosky et al. [18] address learning of conceptual knowledge (‘what’). During medical studies, applying procedural knowledge (practical skills, ‘how’) is essential as well [24] and considers other techniques, for example practical learning in the skillslab or workplace. In further research, it could be explored which other learning techniques are used by students.

Our model explains approximately 10% of the variance in perceived difficulties, which means that there must be various other reasons that have an impact on the experienced difficulties. These could be exogenous factors, such as the students’ medical education context regarding living arrangements, the level of social support, work besides their studies, financial concerns, etc. [28].

In further studies, the relationship between the learning techniques used by students and exam scores should be investigated. Moreover, further research should cover the relationships between the different learning techniques used to explore how high-utility learning techniques are connected with low and moderate learning techniques.

## Conclusions

On the one hand, medical students are responsible for their own academic achievement; on the other hand, teachers have a major influence on the success or failure of their students [5]. This work may help students rethink their learning techniques and utilize their full potential for their examination performance; however, it can also invite medical teachers to better inform students about effective learning techniques. Faculties can support students’ learning by implementing information about learning opportunities in the curriculum. Learning how to learn should also be part of the curriculum, and students should be informed about the different utilities of varying learning techniques. The results may help students and teachers facilitate students’ learning and academic success.

## Supplementary Information

**Additional file 1.**

## Data Availability

The dataset used and analysed during this study are not publicly available due to ethical and regulatory restrictions is available from the corresponding author on reasonable request.

## Abbreviations

Charité: Charité – Universitätsmedizin Berlin
MCM: Modular Curriculum of Medicine
