# Supplementary material for: How do medical students learn conceptual knowledge? High-, moderate- and low-utility learning techniques and perceived learning difficulties
Source: BMC Med Educ. 2022 Apr 6;22:250. doi: 10.1186/s12909-022-03283-0 (PMC8988315; doi:10.1186/s12909-022-03283-0)
Supplement: Supplementary file 1 — Additional file 1. [file 12909_2022_3283_MOESM1_ESM.pdf]

Appendix

Items from the standardized questionnaire used to capture the agreement of students regarding the use of common learning techniques according to Dunlosky et al. [18].

**I use the following learning techniques to learn:**<br>*1 = strongly agree; 2 = agree; 3 = neutral; 4 = disagree; 5 = strongly disagree*
**Elaborative Interrogation**[2]<br>No mere memorization of information, but rather explicit questioning why a fact or concept is correct
**Self-explanation**[2]<br>Finding links, how a new piece of information is related to already known information, or which steps to solve a problem or a task are necessary
**Summarization**[1]<br>Summarizing key statements of a text
**Highlighting/Underlining**[1]<br>Marking important passages in the text
**Keyword Mnemonic**[1]<br>Learning with the help of mnemonic devices, e.g. mnemonic sentence, rhyme, diagram
**Imagery for Text**[1]<br>Producing real sketches or mental images of specific learning content
**Rereading**[1]<br>Repeated reading of a text
**Practice Testing**[3]<br>Solving of tasks related to the content you have learned, e.g. cognitive content by means of quizzes or multiple choice questions, or practical content through trial and error
**Distributed Practice**[3]<br>Dividing the content to be learned into small units to learn this "in portions" over a certain period of time
**Interleaved Practice**[2]<br>Within a learning session, different problems, topics or learning materials are mixed

Legend: [1]Low utility, [2]Moderate utility, [3]High utility
